# Supplementary material for: Construction of Electrochemical Immunosensor Based on Gold-Nanoparticles/Carbon Nanotubes/Chitosan for Sensitive Determination of T-2 Toxin in Feed and Swine Meat
Source: Int J Mol Sci. 2018 Dec 5;19(12):3895. doi: 10.3390/ijms19123895 (PMC6320875; doi:10.3390/ijms19123895)
Supplement: Supplementary file 1 [file ijms-19-03895-s001.pdf]

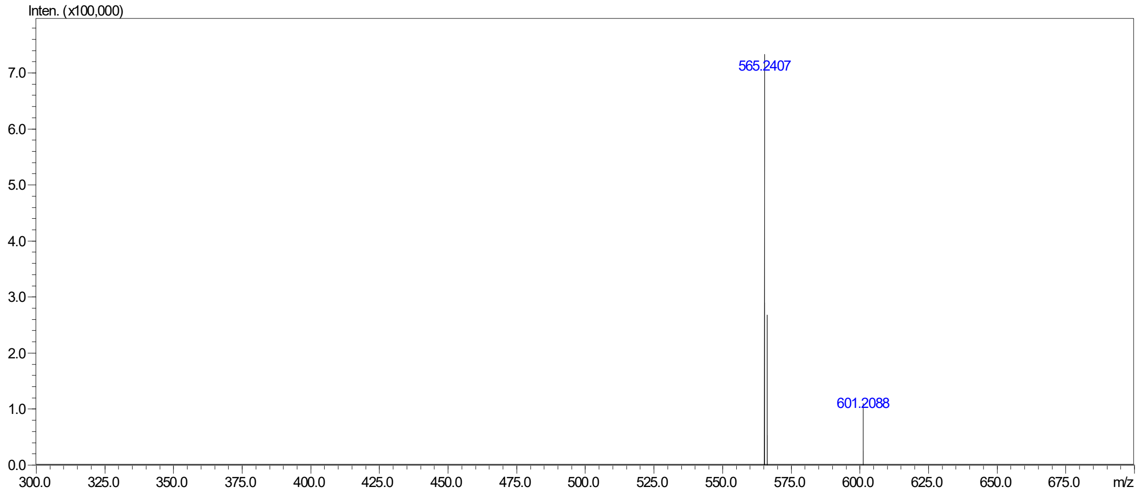

Figure S-1 The mass spectrum of t-2 hapten.

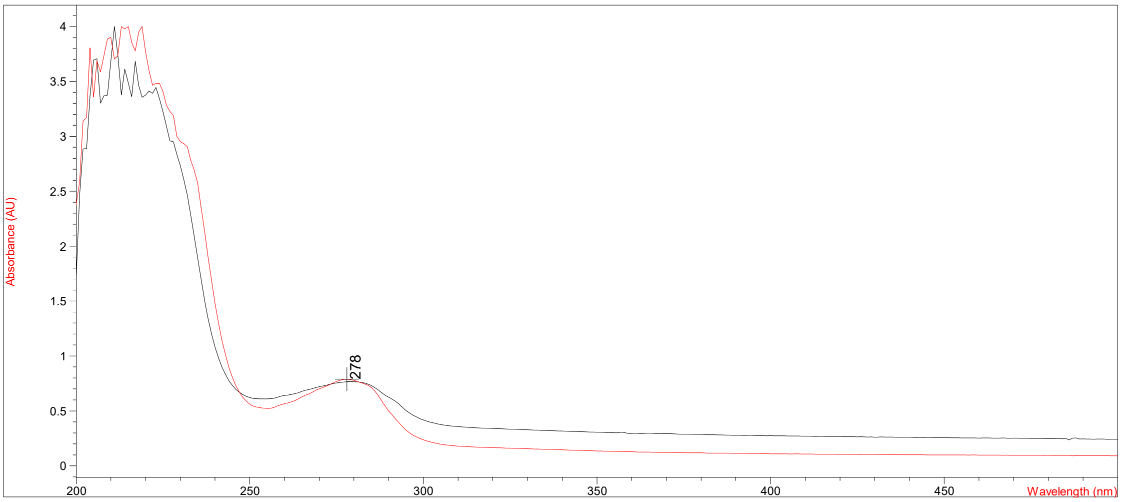

Figure S-2 The UV-Visible spectra of t-2 antigen.

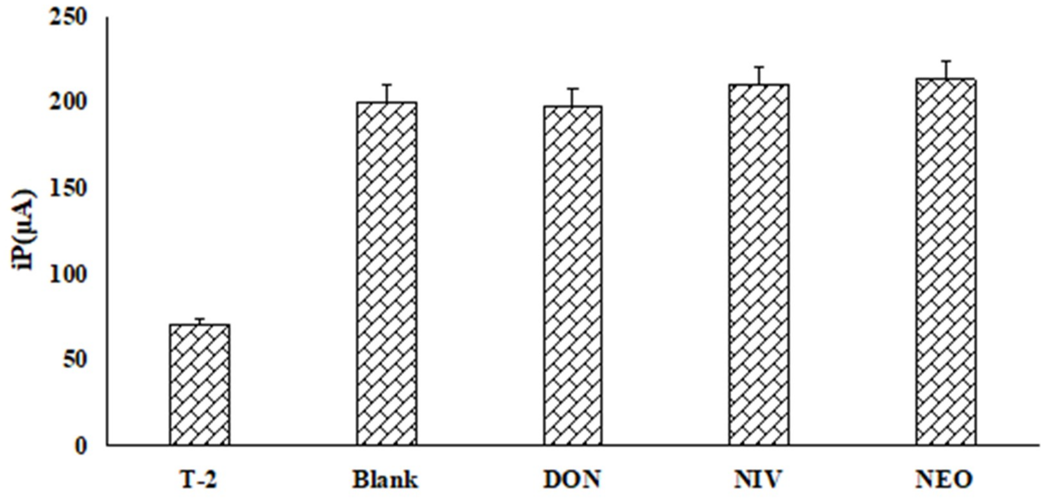

Figure S-3 The specificity comparison result of the immunosensor.

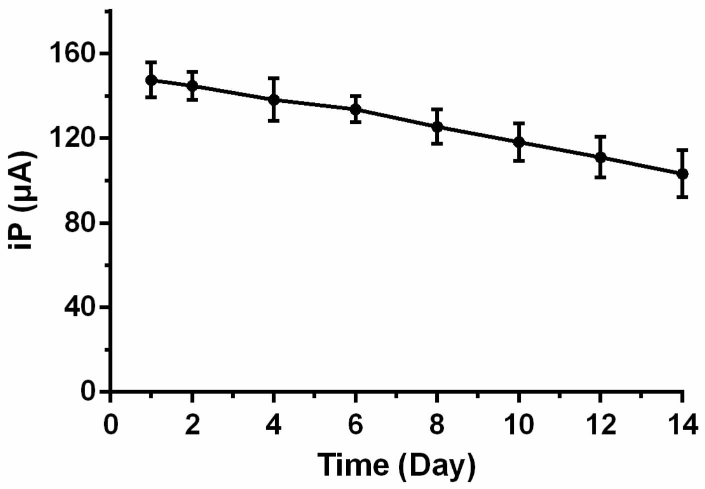

Figure S-4 The immunosensor stability result.
